# Informal value chain actors’ knowledge and perceptions about zoonotic diseases and biosecurity in Kenya and the importance for food safety and public health

**DOI:** 10.1007/s11250-017-1460-z

**Published:** 2017-11-13

**Authors:** Simon Nyokabi, Regina Birner, Bernard Bett, Linda Isuyi, Delia Grace, Denise Güttler, Johanna Lindahl

**Affiliations:** 1 Wageningen University, Wageningen, Netherlands; 2 Universität Hohenheim, Stuttgart, Germany; 3 International Livestock Research Institute, Nairobi, Kenya; 4 Swedish University of Agricultural Sciences, Uppsala, Sweden

**Keywords:** Biosecurity measures, Zoonoses, Livestock value chains, Veterinary public health, Disease prevention, Infectious disease, Epidemiology, Disease transmission

## Abstract

Zoonotic diseases, transmitted from animals to humans, are a public health challenge in developing countries. Livestock value chain actors have an important role to play as the first line of defence in safeguarding public health. However, although the livelihood and economic impacts of zoonoses are widely known, adoption of biosecurity measures aimed at preventing zoonoses is low, particularly among actors in informal livestock value chains in low and middle-income countries. The main objective of this study was to investigate knowledge of zoonoses and adoption of biosecurity measures by livestock and milk value chain actors in Bura, Tana River County, in Kenya, where cattle, camels, sheep and goats are the main livestock kept. The study utilised a mixed methods approach, with a questionnaire survey administered to 154 value chain actors. Additional information was elicited through key informant interviews and participatory methods with relevant stakeholders outside the value chain. Our results found low levels of knowledge of zoonoses and low levels of adherence to food safety standards, with only 37% of milk traders knowing about brucellosis, in spite of a sero-prevalence of 9% in the small ruminants tested in this study, and no slaughterhouse worker knew about Q fever. Actors had little formal education (between 0 and 10%) and lacked training in food safety and biosecurity measures. Adoption of biosecurity measures by value chain actors was very low or non-existent, with only 11% of butchers wearing gloves. There was a gendered dimension, evidenced by markedly different participation in value chains and lower adoption rates and knowledge levels among female actors. Finally, cultural and religious practices were shown to play an important role in exposure and transmission of diseases, influencing perceptions and attitudes to risks and adoption of biosecurity measures.

## Introduction

In many developing countries, animal-source food is an important source of nutrition for consumers and an important source of livelihood for value chain actors. Livestock and their products are a source of zoonotic infections as most human diseases are also animal diseases (Greger 2007). Livestock value chains act as important pathways for transmission and spread of zoonosis (FAO 2011), and higher sero-prevalence of zoonoses among actors in livestock value chains compared to the general population is often reported, possibly due to occupational exposure to zoonotic pathogens (OIE 2006).

Relatively cheap and cost-effective biosecurity measures could reduce the negative impacts of zoonoses (FAO 2008), by ensuring ‘the exclusion, eradication or effective management of risks posed by pests and diseases to the economy, environment and human health’ (Frampton 2010; page 4). However, as intervention has to date focused on farm rather than value chain level (Schelling et al. 2007), adoption of biosecurity measures has been low (Schelling et al. 2007). This is increasingly considered a major public health problem (Ngasala et al. 2015).

As research has primarily been undertaken in a developed country context (FAO 2008); knowledge is lacking as regards adoption of biosecurity measures in developing countries (Roesel and Grace 2014). Although most biosecurity measures can be applied in a wide range of contexts, a tailor-made approach is needed as a ‘one size fits all’ is not the ideal model to transfer knowledge and practices to developing countries (Omore et al. 2001), which means that it is important to assess a situation locally before suggesting interventions.

This paper focuses on meat (small ruminants [sheep/goats] and cattle) and milk (cattle and camel) value chains in Kenya and draws on the results of a study which took place in the context of the ‘Dynamic Drivers of Disease in Africa’ project which looked at Rift Valley fever (RVF) and vector-borne diseases in Kenya. Despite the known occurrence of both epidemic and endemic diseases in the study area—slaughtering, skinning and other contacts with animal body fluids is a risk factor for RVF transmission to humans (LaBeaud et al. 2008; Anyangu et al. 2010), and although often underdiagnosed, and the extent of its burden and epidemiology is unknown, brucellosis causes severe disease in humans and animals (Boschiroli et al. 2001; Akakpo et al. 2010; Alonso et al. 2016)—how value chain actors viewed biosecurity measures and how knowledgeable they were about the risks of zoonoses in the study area were not well known.

The objective of this study was to explore value chain actors’ knowledge and understanding of zoonotic risks, to assess their perception of the significance of identified zoonotic risks and their incorporation of biosecurity measures in their daily activities and workplaces, and finally, to identify the factors influencing the levels of adoption of biosecurity measures of different value chain actors. Arguing that informal value chains will continue to exist and dominate in developing countries at least into the near-term future, this paper suggests that value chain actors’ adoption of biosecurity measures could play a critical role in improving food safety and in public health efforts to reduce future transmission and spread of zoonoses and food-borne illnesses.

## Methodology

### Study area

**Fig. 1:**
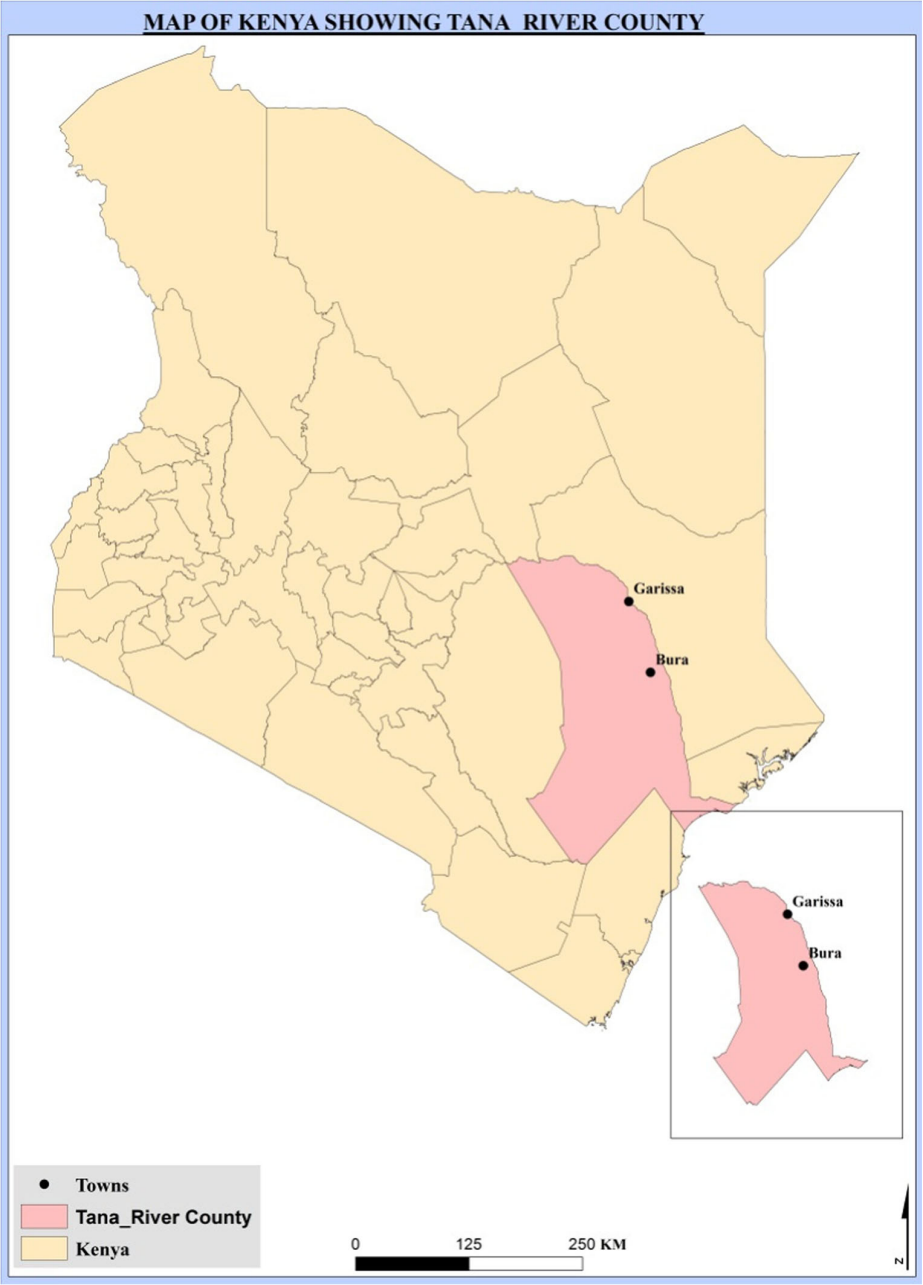
Map of Bura, Tana River County in Kenya

The study was conducted in Bura town, Tana River County (Fig. 1), in a region populated by pastoralists and agro-pastoralists (Munyua et al. 2010; Sang et al. 2010). Recent establishment of irrigation schemes in the region has led to land-use change; increasing wildlife, livestock and human interaction; and conditions favourable to insect vectors and vector-borne zoonoses (Sang et al. 2010).

### Methodological approach

A mixed methods approach (Brannen 2005) was taken to assessing the adoption of biosecurity measures in livestock trade, meat and milk value chains. A questionnaire was administered to 154 value chain actors (livestock traders, milk traders, abattoir workers and transporters) in Bura market by trained enumerators in Kiswahili (Table 1). Diseases and their symptoms both in humans and animals were discussed using local and Swahili names which were validated prior to the start of the study. These diseases were chosen because of the high socio-economic implications for value chain actors and their symptomatic similarity to common diseases such as malaria. In the context of this study, biosecurity measures were defined as any measures employed by value chain actors to prevent transmission of zoonoses and other infectious diseases, and to ensure food safety. Four categories of biosecurity measures were investigated: personal, environmental, food safety and animal health. A matrix of biosecurity measures was generated (Table 6) comparing cost versus ease of implementation.

**Table 1:** Composition of study sample (value chain actors) in a study on biosecurity measures in Tana River County, Kenya

		Traders	Butchers	Transporters	Slaughterhouse workers	Milk traders
Sample size		43	9	35	10	57
	Male	95.3%	88.9%	97.1%	100%	7%
	Female	4.7%	11.1%	2.9%	–	93%
Mean age		42.86	38.56	32.17	38.20	32.11

Results were disaggregated by value chain group to account for actors being subject to different laws and regulations and having differing incentives to adopt biosecurity measures. The study sample was selected using purposive and snowball sampling techniques (Onwuegbuzie and Collins 2007) as there was no exhaustive list of value chain actors. We also ensured inclusion of traders, who mostly operated only on market days. Due to actors’ time constraints, rather than adhering to classical focus group methodology, six loosely structured informal group discussions (comprising four to six participants) were held in Kiswahili on issues relevant to the topic of the study. These discussions were recorded with permission of the actors and transcribed and translated to English for content analysis. Direct observation (Lundy et al. 2008) was undertaken daily for 28 days at two slaughterhouses, a livestock market and business premises.

Participatory mapping was used to map the value chains, geographical locations of activities undertaken and the role of different actors, stakeholders and important institutions in the functioning of the value chains. This facilitated identification of critical points of risk for zoonoses transmission and spread in the value chains, and opportunities existing to improve food safety issues (Table 5). A powerful tool for visualisation, participatory mapping is cheap and requires little technical knowledge and allows participants to be active rather than passive in knowledge generation (Bett et al. 2009). Key informants were interviewed, with a total of 13 relevant stakeholders from and beyond the value chain selected based on their experience and knowledge (two participants for livestock traders, slaughterhouse workers, milk traders, transporters, butchers, veterinary/public health officials; and one human health practitioner).

Blood samples were collected in vacutainers from small ruminants prior to slaughtering and centrifuged and stored in cold ice, for transport to Nairobi. They were analysed using a competitive ELISA (enzyme-linked immunosorbent assay) test for *Brucella* antibodies (SVANOVIR, Uppsala Sweden), following the manufacturer’s protocol.

### Data management

Quantitative data collected through a cross-sectional survey was entered into Microsoft Excel and cleaned. Descriptive statistics were performed using SPSS version 9.0 (SPSS, Inc., Chicago, IL). Statistical testing was undertaken using *χ* ^2^. Qualitative data collected through recorded informal discussions and key informant interviews was transcribed, translated and subject to content analysis. Participatory maps were re-drawn in Microsoft Word to ensure clarity. Data collected in this project was stored and made available as per the data management and sharing policies of Hohenheim University and ILRI.

## Results

Participation in livestock value chain activities is dictated by gender. Men participate more in livestock and meat value chain activities, while women participate more in the milk value chain activities. However, while few men participated in the milk value chain, a small number of women were meat traders, butchers or transporters. No women were found to work in the slaughterhouse. Consequently, women accounted for 37% of the study sample. Actors’ understanding of occupational risks was low, as was their concern over biosecurity and their knowledge of zoonoses in both humans and animals (Table 2). Most actors were familiar with brucellosis (55%), RVF (68%) and cysticercosis (88%), and least familiar with Q fever (9%), leptospirosis (34%), salmonellosis (36%) and anthrax (41%) (Table 2). Most actors could not differentiate taeniasis from cysticercosis, or leptospirosis from bilharzia, due to their similar symptoms.

**Table 2:** Value chain actors’ knowledge of zoonoses and biosecurity in Tana River County, Kenya

	Livestock traders, *n* = 43 (%)	Butchers, *n* = 9 (%)	Transporter, *n* = 35 (%)	Slaughterhouse workers, *n* = 10 (%)	Milk traders, *n* = 57 (%)
Heard about zoonoses	72	78	66	90	47
Know how to protect yourself from zoonoses	56	44	49	90	37
Think biosecurity measures are important	58	44	49	90	37
Know brucellosis	67	57	70	44	37
Know tuberculosis	61	40	90	44	40
Know anthrax	28	46	90	44	19
Know rabies	47	43	80	56	33
Know salmonellosis	42	40	30	44	32
Know cysticercosis	98	77	100	89	74
Know Rift Valley fever	88	71	90	67	40
Know Q fever	14	17	10	0	4
Know leptospirosis	40	37	50	33	19
Have formal training	2	0	0	10	2
Have on job training	16	44	29	70	7
Have no training	81	56	71	20	91

### Personal biosecurity

The study results indicate low levels of education among value chain actors, with over 90% reporting no formal/academic education (including courses or official training). Slaughterhouse workers were the only group who reported formal or on-the-job training. Women reported less on-the-job training than men. Many actors lacked training on food handling and occupational risk reduction (Table 2). Results show that 74% of transporters, 86% of traders and 84% of milk traders had never undergone mandatory medical check-ups required for food handlers. Only butchers and slaughterhouse workers reported regularly receiving medical check-ups as a prerequisite for being granted a working certificate/permit. Female actors reported lower rates of annual medical examination (*p* = 0.038) compared to their male counterparts. There was a strong association between gender and knowledge of zoonoses (Table 3), with lower knowledge among female actors (*p* = 0.017), and an association between gender and use of protective clothes (*p* = 0.001), with female actors having lower adoption rates.

**Table 3:** Chi-square comparison of gender with adoption of selected biosecurity measures

	Male *n* = 97		Female *n* = 57			
	No	Yes	No	Yes	*χ* ^2^	*P*
Knowledge of zoonoses	29 (30%)	68 (70%)	28 (49%)	29 (51%)	5.6924	0.017
Use recommended protective clothes	68 (70%)	29 (30%)	54 (95%)	3 (5%)	13.2348	0.000
Had annual examination	63 (65%)	34 (35%)	11 (19%)	46 (81%)	4.3080	0.038

Discussions revealed low levels of adherence to regulations regarding public health and food safety as most actors operated outside public health inspectors working hours (8 A.M.–4 P.M.). Low level of enforcement by authorities was linked to low staffing capacity, low pay, lack of transport and other challenges (Table 5). According to Kenyan law, the use of personal protection equipment (PPE) by those handling food is mandatory to prevent the risk of contamination. However, direct observations revealed low adoption by all value chain actors, with some actors choosing not to use PPE and others only partially using PPE (Table 4). Actors reported perceiving use of PPE as cumbersome, reducing their productivity and as generating no tangible benefits.

**Table 4:** Percentage of livestock actors observed using different recommended personal protective equipment in Tana River County, Kenya

	Traders, *n* = 43 (%)	Milk traders, *n* = 56 (%)	Transporters, *n* = 35 (%)	Butchers, *n* = 9 (%)	Slaughterhouse workers, *n* = 10 (%)
Aprons and overalls	16	0	0	89	90
Gumboots	7	0	20	22	90
Gloves	2	0	14	11	70
Head covering	0	0	6	22	70

### Food safety

Livestock traders reported undertaking visual inspections of live animals, while meat was inspected by the public health department before it was released for transport from the abattoir to butcheries for sale. The study found 9% of serum samples collected from animals due to be slaughtered as testing positive for *Brucella* antibodies.

Milk traders (97%) reported undertaking quality tests on milk, including tasting (taking a sip of raw milk), 77%; observing the colour, 18%; checking milk butter content, 11%; and conducting the clot-on-boiling test, 12%. Untreated water was frequently used for cleaning and washing, and water was purchased from vendors who had sourced it from irrigation canals when slaughterhouse water tanks were empty. Value chain actors reported distress sales and slaughtering sick animals to avoid losses. The reasons given for slaughter of sick animals included home consumption, not being aware that slaughtering was inadvisable, to avoid costs of animal disposal, to comply with cultural beliefs and to earn money. Animals slaughtered in the homestead were never inspected by officials prior to consumption and meat was often shared with neighbours.

### Food packaging

Milk from different origins was bulked in larger containers. Observations showed that traders stored milk in plastic containers, while meat was hung in the open, without protection from dust or flies. Traders did not refrigerate meat or milk overnight, despite the risks of quick spoilage or deterioration of quality in the hot and humid study area. Butchers reported selling meat wrapped in old newspaper (22%) and/or wrapped in polythene first then an old newspaper (67%).

Milk was sold packaged in polythene paper (74%), or in recycled plastic bottles (56%) which were not properly cleaned or sterilised. Although some vendors kept milk in open containers (3.5%), it was more commonly kept in closed containers (74%). Most traders (60%) boiled milk to extend shelf life. Direct observations revealed unhygienic handling of containers used for transporting milk and meat boxes, exposing them to dust, flies and other sources of contamination. No actors reported sterilising their containers after or before use, and many reported washing them using soap/detergent powders and untreated water from irrigation canals.

### Animal health biosecurity and biosecurity failures

Animal health biosecurity measures observed by livestock traders (*n* = 43) included spraying livestock for vector control (86%), isolating livestock at the market (56%), inspecting livestock at the markets (54%), quarantining livestock at the markets (40%) and (35%) reporting when livestock died at the market. When animals died, the actors reported that they burned the carcass (26%), buried the carcass (16%), reported livestock death immediately to vet (2%) and disposed the carcass in the open (7%) (for scavengers to eat). Some reported that they used (consumed) the dead animal (9%). Livestock traders reported treating sick animals with veterinary drugs obtained over-the-counter, often without advice from veterinary officers. Some traders and livestock keepers used medicine intended for humans to treat sick animals. In cases where the market committee (managerial group selected by traders) detected sick animals (through visible symptoms), animals were treated by a veterinary officer and the owner was advised to take them back home until the disease was gone. However, there was no strict enforcement of this directive, and therefore, best practices regarding treatment and isolation of sick animals were not observed by all actors.

### Environmental biosecurity

The disposal of waste and carcasses did not meet the standards envisaged by the legislation in Kenya. Direct observations suggested that people bringing cattle to market, typically left dead animals behind without burying or burning them, occasionally using twigs or tree branches to cover the carcasses, while traders buried animals which died during transport, or burned them with old car tyres. These deaths were not reported to authorities.

Slaughterhouses were observed to dump waste in the open as septic tanks were full, attracting scavengers such as dogs and chickens, while dogs (usually non-vaccinated) were observed to wander around the slaughterhouse and in the vicinity of an open garbage place where milk was traded. The cultural practice of open defecation and low use of latrines by the local population constituted an additional public health risk (Table 5). Facilities such as slaughterhouses, milk bulking areas and markets lacked hand-washing facilities, and while most butcheries had hand-washing facilities, the water typically came from irrigation canals and was therefore likely contaminated.

### Cultural and religious issues

Consumption of undercooked meat, raw meat and milk, and meat and milk derived from sick animals was common. Moribund animals were slaughtered for consumption by the predominantly Muslim population (who do not consume dead animals) in the study area. The reasons given by respondents for slaughtering sick animals included consumption (49%), ignorance (19%), to avoid animal disposal costs (16%), to ensure cultural beliefs were met (9%) and to sell the meat (2%). Religious beliefs shaped perceptions of diseases as some actors reported said there was no need to prevent disease transmission and infection as they had divine protection and could rely on prayers to heal disease. Conversely, some value chain actors believed that disease outbreaks were a divine punishment. Cultural values and practices also influenced health-seeking behaviour of individuals as some individuals opted to seek divine cure through prayers first, only seeking health care intervention in the advanced stages of disease.

Pastoralists reported eating sick animals suffering from anthrax disease (*chimale*/*chirrmalle in Gabbra language*), saying that they believed they could derive protection from disease by tying the bark of a local shrub around their wrist. Some actors reported not going to hospital when they subsequently suffered from *chimale* as they believed getting an injection would lead to death. In many cases, they waited until the boil-like lumps burst before going to hospital to seek medical attention, sometimes when it was very late.

**Table 5:** Suggested critical points, legislation involved and potential interventions resulting from interviews and discussions with informal value chain actors in Tana River County, Kenya

Actor	Critical points	Legislation	Potential intervention
Livestock traders	• Buying animals without testing • Open defecation • Manure and waste management • Visual inspection of animals	• Animal movement certificate • Animal trading licences	• Animal quarantine • Thorough animal inspection • Proper dead animal disposal
Milk traders	• No PPE used • Unhygienic handling • Non sterilisation of equipment and plastics • Lack of medical exams • Handling milk in open grounds	• Business licence • Public health certificate • Medical certificate	• Enforcing medical certificate requirement • Construction of work stalls • Training • Provision of credit to purchase pasteurising equipment
Slaughter house workers	• No PPE used • Unhygienic handling • Non sterilisation of equipment • No regulations • Untreated water • Raw milk and offal consumption • No certification programs	• Public health certificate • Medical certificate	• Training actors • Provision of clean water • Enforcing medical certificate requirement
Transporters	• Trekking animals • Lack of animal testing • Poor disposal or carcases	• Animal movement certificate • Milk movement certificate	• Training actors • Proper cleaning of vehicles • Testing animals before transporting them
Butchers	• Non sterilisation of equipment and plastics • Lack of medical exams	• Business licence • Public health certificate • Medical certificate	• Training actors • Provision of credit to purchase cooling/storage equipment • Enforcing medical certificate requirement

### Cost versus ease of implementation of preventive biosecurity measures

Discussion with value chain actors led to development of a matrix of biosecurity measures **(**Table 6), which could be adopted based on their costs and ease of implementation. Actors were most interested in practical interventions, which were not costly and would not be time-consuming to implement in the process of undertaking their daily activities. They reported being distrustful of interventions which required extensive level of knowledge and skills, or a large investment of resources, given the small profit margins associated with operating in markets characterised by low prices for milk and meat.

**Table 6:** Matrix of biosecurity cost versus ease of implementation resulting from interviews and discussions with informal value chain actors in Tana River County, Kenya

	Ease of implementation		
Cost of implementation	Easy—expensive	Somehow easy—expensive	Difficult—expensive
	Testing for diseases	Vaccinations	Sewer systems
	Isolation of animals	Cooling facilities	Testing labs
	Quarantine facilities	Pasteurisation	Good infrastructure
	Public education	Sterilisation of milk (in bottle)	Good governance and
		UHT (ultra-high-temperature) treatment	Laws and policies
		Institutional capacity	Competent body of inspectors (veterinarians, meat inspectors)
		Certification	Testing and culling
	Easy—medium cost	Somehow easy—medium cost	Difficult—medium cost
	Protective clothing	Toilets	New food laws
	Meat inspection	Public education	Testing equipment
	Refrigeration	Food testing	Animals tracing
		Aluminium milk containers	
	Easy—cheap	Somehow easy—cheap	Difficult—cheap
	Washing hands	Medical check ups	Manure disposals
	Disinfection	Licencing	Low-cost packaging
	Water treatment	Ante-mortem inspection	
	Sanitation use	Post-mortem examination	
	Premises inspections		

## Discussion

An RVF outbreak of 2006–2007 in Tana River County highlighted the negative socio-economic impacts of zoonoses in Kenya’s regions dependent on informal livestock value chains (Anyangu et al. 2010; Munyua et al. 2010). The results of this study highlight how low level of knowledge of zoonoses, disease symptoms and modes of transmission among value chain actors may contribute to higher occupational risk (Aburi 2012). Similar to conclusions reached by Mwangi et al. (2000), low adoption of biosecurity measures can be linked to actors’ lack of training and low levels of education. The findings of this study indicate that slaughterhouse and traders are exposed to actual risks as 9% of the small ruminants slaughtered tested positive for antibodies against *Brucella* bacteria.

The results of this study, similar to Leksmono et al. (2006), highlight the challenges faced by informal value chain actors in accessing treated water, electricity, training, skills, tools and other resources. Although traders saw the need to control milk quality, the tests undertaken were not scientific, had low accuracy and comprised a risk for the trader. Lack of equipment to test milk quality directly exposed value chain actors to diseases. Tests including sipping untreated milk can lead to disease transmission and cross-contamination of the milk posing a health risk to the greater population, and they fail to detect presence of chemical or microbial contaminations or adulteration. The cleaning of milk containers, as practiced by the actors, is not sufficient to eliminate microbes, and rinsing with unclean water may introduce new pathogens, and similar to findings of Grace (2011), this study found most actors operated in unhygienic conditions which increased the probability of contamination during food processing and handling.

Chengula et al. (2013) and Grace et al. (2012) observe that poor and vulnerable segments of society bear the greatest burden of zoonotic diseases. Value chain actors have a responsibility to manage risk and prevent contamination as they benefit socially and economically from activities (Ngasala et al. 2015). Their reluctance to voluntarily and rigorously follow regulatory directives may be due to lack of knowledge of the health and economic benefits of adopting biosecurity measures and a belief that adoption costs may exceed benefits, as well as poor enforcement of laws by officials.

There is a gender dimension to zoonotic disease risk and exposure, but this study cannot verify the conclusions reached by a study undertaken in Nigeria, which found that women were more conscious and observed higher levels of hygiene than their men counterparts (ILRI 2011). Findings that female actors lagged behind in this study—as regards adoption of biosecurity measures—could be explained by the fact that women face greater challenges than men in accessing credit and lack the knowledge, skills and collateral required to access financial markets (Coles and Mitchell 2011), highlighting the need for gender empowerment to improve value chain actors’ livelihoods and access to safe food. While this study did not use a random sampling, gender differences in the participation in different activities were apparent, and unlikely to be due solely to selection bias.

This study identified policy and governance constraints, which need to be addressed to improve food security and adoption of biosecurity measures by value chain actors. In many developing country contexts, policies are made by rent-seeking individuals without the consensus of all stakeholders leading to problems in implementation and enforcement (Leksmono et al. 2006). As noted by Kutalek et al. (2015), pro-poor policy and a risk-based approach to controlling the transmission and spread of zoonoses is needed to ensure the success of laws, and the successful policies and interventions are contingent on all stakeholders being involved in the design process. Government entities play an important role in food safety issues and also in shaping actors’ perception and understanding of biosecurity measures. There is a need to involve actors in enforcement of laws to ensure voluntary uptake of beneficial measures and reduce the need for strict supervision (Leksmono et al. 2006; Kutalek et al. 2015). The authors believe that actors could, if required, organise themselves to implement measures to safeguard their businesses, for example, preventing the trade of sick animals, to comply with trade bans and quarantine directives.

The results of this study confirm the findings of Roesel and Grace (2014) that there is a need for policy to take into consideration religious, social and cultural issues which influence individuals’ action-taking and decision-making, including their health care-seeking behaviour. This study revealed that some value chain actors believe that disease outbreaks are a divine punishment, while some rely on prayers to heal rather than seeking timely medical attention.

Non-reporting of animal disease incidences combined with undocumented movements (without issued livestock movement certificates) poses a challenge to Kenya’s national veterinary services, undermining their capacity to intervene in the initial aftermath and crucial time period following a disease outbreak to halt the transmission and spread of zoonoses. The results of the study suggest that there is a need to devise new methods to encourage reporting of animal deaths and to reduce sale or slaughter of sick and moribund animals. For example, cash compensation for mandatory culling could discourage distress sales of sick animals (Gunn et al. 2008; Toma et al. 2013).

The results of this study confirm the findings of Omore et al. (2001) that there is often marginalisation and victimisation of actors who participate in informal markets, which according to Grace (2011), exacerbates low hygiene and undermines adoption of biosecurity measures. As observed by Leksmono et al. (2006), resource-poor actors often cannot justify and afford to invest in innovations and measures which they regard as vague and generating no tangible benefits. It is paramount to understand that adoption is driven by access to capital, education levels, training, cost and ease of implementation, legal environment and by the benefits perceived as derived from adopting innovations (Grace 2014). Moreover, actors may not attain or see direct benefits of adopting biosecurity measures, which reduces their willingness to practice them.

Value chain actors are reluctant to invest in adopting measures voluntarily. Leksmono et al. (2006) recommends harmonisation and enforcement of already existing legal framework. Innovations including licences could be used to link informal and formal markets although progress has been slow (Leksmono et al. 2006). In addition, Roesel and Grace (2014) argue that formal value chains are not necessarily safe and informal value chains are not necessarily unsafe, showing the importance of assessing the actual risk rather than hazards present in informal value chains.

To date, developing countries have not sufficiently invested in research, institutions, policies and infrastructure in informal value chains (Leksmono et al. 2006; Onono et al. 2013). Understaffing and underfunding of agencies tasked with food safety and public health hinder execution of their mandates (Grace 2014). Privatisation has hindered access to free or subsidised government veterinary services in resource-poor communities, while on the other hand, it can be argued that it has led to increased access to extension and improved animal health through self-treatment or provision of services by non-veterinary providers (Onono et al. 2013). Given that reduction of poverty is a public good (Schelling et al. 2007), there is justification for investment in public health to control transmission and spread of zoonoses.

These findings reinforce the need for a farm-to-fork framework such as ‘One Health Initiative’ for investing public money to control zoonoses as it is cost-effective and generates multiple health benefits (Grace 2014). There is need for a clear strategy for policy-makers and practitioners to guide the design and implementation of policies and initiatives aimed at controlling zoonoses prevalence, transmission and spread within informal value chains (Aburi 2012).

Finally, it is worth noting that it is hard to generalise these findings or to even draw conclusions based on statistical inference as sample size was small and not randomly selected; however, they still provide a hypothesis that can further be explored in subsequent studies.

## Conclusion

The results of the study highlight the need to involve value chain actors in the design and implementation of food safety, biosecurity measures and policies. Improving food safety standards in developing countries with scarce resources can generate additional benefits including food security, a reduction in the number of deaths among children under 5 years and immune-compromised individuals, and improved livelihoods and increased savings associated with improved health status. There is an urgent need to improve adoption of biosecurity measures in developing countries where the marginal in society, engaged in or dependent on informal value chains for their livelihood, bear the burden of zoonoses and related adverse impacts.
